# Molecular Mechanisms of Induced Pluripotency

**Published:** 2012

**Authors:** I.A. Muchkaeva, E.B. Dashinimaev, V.V. Terskikh, Y.V. Sukhanov, A.V. Vasiliev

**Affiliations:** Koltzov Institute of Developmental Biology, Russian Academy of Sciences

**Keywords:** reprogramming, iPS cells, ES cells, differentiation, transformation, pluripotency

## Abstract

In this review the distinct aspects of somatic cell reprogramming are discussed. The molecular mechanisms of generation of induced pluripotent stem (iPS) cells from somatic cells via the introduction of transcription factors into adult somatic cells are considered. Particular attention is focused on the generation of iPS cells without genome modifications via the introduction of the mRNA of transcription factors or the use of small molecules. Furthermore, the strategy of direct reprogramming of somatic cells omitting the generation of iPS cells is considered. The data concerning the differences between ES and iPS cells and the problem of epigenetic memory are also discussed. In conclusion, the possibility of using iPS cells in regenerative medicine is considered.

## INTRODUCTION

Pluripotent stem cells are capable both of self-renewal and generation of all the cell types that constitute the three germ layers. Until recently, pluripotent stem cells were derived from cultures obtained from the internal cell mass of the blastocyst (embryonic stem cells – ESCs) [1, 2]. However, the procedure of obtaining ESCs was burdened with numerous practical and ethical issues that made it impossible to use ESCs in clinical practice. Because of this, the global scientific community pressed on with its active search for an appropriate method for obtaining cells with characteristics similar to those of ESCs. Certain progress was achieved in 1997, when Wilmut *et al* . reprogrammed breast somatic cells via the transfer of their nuclei into oocytes after the second meiotic division (somatic cell nuclear transfer, SCNT) [3–6]. In 2001, Tada *et al* . achieved the same result via the fusion of mouse thymocytes with ESCs [7]. However, all attempts aimed at eliminating the technical complexity and low reproducibility of these methods failed, as did the attempts aimed at using these techniques for primate cells.

In 2006, based on accumulated data, Takahashi and Yamanaka assumed that an unfertilized cell and ESCs contain pluripotency-determining factors [8]. The method for the introduction of genes playing a key role in early development using lentiviral constructs was described in their studies on mouse fibroblasts [8] and, subsequently, on human cells [9]. It was successfully demonstrated that the ectopic gene expression of only four transcriptions factors, Oct4, Sox2, Klf4, and c-Myc (subsequently referred to as the KMOS canonical gene set, or the “Yamanaka cocktail”), is sufficient for the reprogramming of fibroblasts into a pluripotent state. The cells obtained using this procedure were referred to as induced pluripotent stem cells (iPSCs); the phenomenon of reprogramming into a pluripotent state was referred to as induced pluripotency. Many characteristics of iPSCs are identical to those of ESCs (e.g., gene expression profiles, morphology, telomerase activity, the character of DNA methylation and histone modification). Furthermore, iPSCs are capable of *in vitro* generation of the tissue cells of the three germ layers; they form mature teratomas after they are injected into immunodeficient mice. Chimeric animals were successfully created; their descendants included the ones obtained from the reprogrammed cells [10, 11]. At the time of writing, a significant number of studies have been published reporting that human iPSCs have been obtained via various methods [12]. Cell reprogramming techniques characterized by higher efficiency and safety compared to the transfection of viral vectors have been designed for potential clinical use [13]. iPSCs from patients with various inherited diseases have been obtained [13, 14]. There are two extensive research areas associated with cell reprogramming: namely, fundamental research of cell plasticity and the genetic mechanisms underlying the early development of the organism and neoplasias, and the technologies for reprogramming somatic cells in order to conduct substitution cell therapy [15]. The cell technologies using iPSCs are capable of providing patient-specific cell lines, including those obtained from the carriers of inherited diseases. These cell lines can be used for the simulation of various diseases and for the testing of new pharmaceutical agents.

## molecular mechanisms underlying pluripotency induction

**Autoregulatory loop. The equilibrium between Klf4 and c-Myc. The impact of the Ink4/Arf locus**

A trove of data has been published to support the hypothesis that pluripotency is regulated by three transcription factors, Oct4, Sox2, and Nanog [16]. It was demonstrated [17, 18] that the combination of Oct4, Sox2, and Nanog factors activates the promoters of both their own genes and the genes of each other, thus forming an autoregulatory loop. Data exists indicating that the autoregulatory loop enhances the stability of the pluripotency gene expression [19, 20]. The three factors under consideration are also capable of initiating the cascades of both active and inactive genes (involving up to several hundreds of them). The expression of the *Oct4, Sox2* , and  *Nanog * genes serves as the basis for the transcriptional network, which ensures the pluripotency of ESCs by enhancing pluripotency gene transcription and simultaneously suppressing the activity of the genes associated with the differentiation and development [21–23].

In their pioneering studies, Takahashi and Yamanaka proceeded with the analysis of 24 genes and subsequently elucidated that four genes ( *Oct4, Sox2, Klf4* and *c-Myc* ) are sufficient for cell transfer into the pluripotent state. Whereas the first two genes are pluripotency master genes, the *Klf4* and *c-Myc * genes were selected for different reasons. The transcription factor с-Myc is known to increase the proliferation rate [24], which is an essential condition for successful reprogramming [25]. Moreover, the hyperexpression of this gene results in an increase in the p53 protein level. It was demonstrated in a number of studies that the expression of *Klf4* leads to an increase in the level of the p21 protein (a cyclin-dependent kinase inhibitor) [23] resulting in proliferation suppression, on one hand, and reduces the cellular level of p53, which has a positive effect on the reduction of the apoptosis risk [26], on the other hand. Thus, one can assume that *c-Myc* and *Klf4* are mutually complementary, their action being oppositely directed. Therefore, equilibrium between the expressions of these two genes is important for successful reprogramming [27].

The inhibition of the *Ink4/Arf* locus, which contains *Cdkn2a* and *Cdkn2b* encoding three powerful tumor suppressors, p16 ( *Ink4a* ), p19 ( *Arf* ), and p15 ( *Ink4b* ), is one of the key characteristics of pluripotent stem cells. It is the *Arf* gene that activates p53 and p21 in mouse cells, whereas the *Ink4a * gene mostly has these functions in human cells. It was demonstrated [28] that the *Ink4/Arf* locus is completely suppressed both in iPSCs and ESCs by epigenetic bivalent domain marks (the emergence of histone modifications repressing H3K27me3); however, the locus can be reactivated upon cell differentiation. Oct4, Sox2, and Klf4 jointly suppress this locus, increasing both the reprogramming kinetics and the number of iPSC colonies, thereby facilitating the enhanced generation of iPSCs. It should also be noted that some researchers directly attribute the activation of the * Ink4/Arf* locus to the overall aging of the organism. Therefore, it is more difficult to reprogram cells taken from an old donor in comparison to those taken from a young one. In this case, the suppression of the *Ink4/Arf* locus can considerably increase the efficiency and rate of reprogramming [25].

**Epigenetic regulation of gene expression in pluripotent stem cells**

ESCs can be distinguished from the differentiated cells due to certain epigenetic characteristics. Thus, the key pluripotency genes ( *Oct4* and *Nanog* ) are demethylated in ESCs and can be actively transcribed, whereas the differentiation results in the suppression of these genes via *de novo* DNA methylation. It is of interest that the methylation marks are removed during reprogramming. This enables the reactivation of the endogenous transcription of these genes [29].

In addition to DNA methylation, ESCs and the differentiated cells also have different histone modification patterns. Thus, the suppression of the genes responsible for the development and differentiation of ESCs is regulated via combinations of the activation (H3K4me3) and repression (H3K27me3) of histone modifications. Transcription regulation is mediated by Polycomb-group proteins, which suppress gene expression by means of their binding to the histones containing H3K27me3. This mechanism is considered to be a tool for the transcriptional flexibility of ESCs, which is conditioned by stable repression of the development-associated genes without the irreversible inactivation of these genes. Taking into account the fact that bivalent domains can be found virtually only in ESCs and are an important characteristic of the pluripotent status, one can assume that the regeneration of “bivalent domains” is also a key stage in the reprogramming of somatic cells in iPSCs. It has been demonstrated in a number of studies that chromatin in the completely reprogrammed iPSCs contains bivalent histones that are identical to those in ESCs [10, 30].

At the time of writing, neither study completely represents the picture of the relationship between the transcription factors, chromatin modifications, and cascades of pluripotency genes during cell reprogramming. Nevertheless, not much data has been published relating to the analysis of the expression of transcription factors in human and mouse ESCs [31] and in iPSCs [32], which can be used as a basis to construct the reprogramming model. Based on the data available, one can assume that Oct4, Sox2, Nanog, and Klf4 are the key units of the reprogramming process, during which nucleosome recovery, assemblage of diacetylases, activation of Polycomb proteins, and chromatin remodeling occur. In 2010, Pereira *et al* . demonstrated in their studies devoted to the fusion of ESCs and lymphocytes that the components of the Polycomb complex play a significant functional role in epigenetic remodeling. ESCs deficient in Polycomb-group proteins lost their ability to remodel the somatic cell genome [33].

The mechanism underlying the suppression of the expression of differentiation genes in pluripotent cells comprises the binding of one or several pluripotency factors to the target gene promoters [34]. The binding between the reprogramming factors and their target genes can be facilitated by nucleosome remodeling complexes, such as Chd1 (chromodomain helicase DNA binding protein 1) [35] and BAF (rg/Brahma-associated factors – АТР-dependent chromatin-remodeling complex) [36]. These complexes improve the reprogramming efficiency and kinetics. Their regulatory role presumably consists in the reactivation and maintenance of the endogenous pluripotency gene expression in the absence of exogenous factors. This assumption is based on the fact that the endogenous pluripotency signals, as well as telomerase and the repressed X-chromosome in female cells, are reactivated by the end of the reprogramming process, whereas the activity of the retroviral genes is suppressed, although no clearly pronounced differentiation and relationship between these processes have been detected [37].

**The role of microRNAs in pluripotency maintenance**

A considerable improvement in reprogramming efficiency after the *Lin28* gene is added to the set of reprogramming factors has been observed in a number of studies devoted to the reprogramming of somatic cells to a pluripotent state [38]. The major contribution of the *Lin28 * gene is thought to be its participation in microRNA (miRNA) processing. It was assumed that in ESCs, *Lin28* inhibits *let7* miRNA processing [39], a well-known tumor growth suppressor gene. This gene participates in the suppression of c-Myc activity. Furthermore, it was demonstrated that the key reprogramming factors Oct4, Sox2, and Nanog are capable of initiating the miR-90 family, whose members are expressed in ESCs under normal conditions and participate in the proliferation regulation, as well as the self-maintenance, of these cells. The activation of mir-290 during reprogramming may also be a result of chromatin remodeling by c-Myc [40]. The miRNA cluster (miR-203–367) promoter is one of the targets of the pluripotency transcription factors Oct4, Sox2, Nanog, and Rex1. Its products can indirectly induce TGF-β/Nodal/Activin signaling pathways (this signaling pathway plays a significant role in the maintenance of the pluripotency status of ESCs and in the suppression of their differentiation) by inhibiting some pathway regulators, which in turn has a positive effect on the maintenance of cells in an undifferentiated state.

**The effect of cell aging and immortalization on reprogramming**

Studies devoted to the interrelationship between reprogramming and the processes of cell aging and immortalization are of considerable interest. It was repeatedly reported in the early studies that telomerase activity increases in somatic cells and telomeric DNA regions last considerably longer in the course of reprogramming. Thus, the reprogrammed cells acquire immortality, typical of ESCs [8, 9, 41]. Yehezkel *et al* . [42] thoroughly studied the telomere length, the methylation in subtelomeric regions, and expression of the telomeric-repeat-containing RNA (referred to as TERRA) in iPSCs. In addition to supporting the previously ascertained data regarding telomerase activation and telomere elongation in iPSCs, it was demonstrated that subsequent differentiation resulted both in a considerable reduction in telomerase expression in the cells under study and in a strong shortening of their telomeric regions. The results obtained in that study provided evidence in support of the significant role of telomerase and the telomere state in the maintenance of the pluripotent status. The subtelomeric regions in iPSCs were hypermethylated compared with the initial cells, whereas the level of TERRA was increased relatively. It is assumed that regulation of the expression of telomeric-repeat-containing RNA can also participate in the reprogramming processes, along with regulation of the expression of the telomerase catalytic component (telomerase reverse transcriptase, TERT) [42].

The essential role of TERRA expression in the reprogramming processes was attested to by the results of study [43], in which the cells of patients with dyskeratosis congenital (a genetic disease associated with telomere dysfunction due to premature telomere shortening) were investigated. Contrary to expectations it turned out that the cells reprogrammed via *Oct4, Sox2, KLf4* , and *c-Myc * also elongate the telomeric regions and restore telomerase activity. The mechanism of restoration of telomerase activity was attributed to the restoration of TERRA expression [43].

Utikal *et al* . demonstrated that the acquisition of immortal status is an essential and limiting factor for the reprogramming of somatic cells into a pluripotent state, which may also attest to the fact that the reprogramming and transformation mechanisms are similar [25].

## THE COMPLEXITY, MULTISTAGENESS, AND stochasticity of pluripotency induction

Low reprogramming efficiency is one of the problems of cell reprogramming through the addition of transcription factors. In the previous procedure of KMOS transfection, approximately 0.01–0.1% of the transfected cells were subjected to reprogramming; this index is considerably lower than that obtained when the cell fusion or nuclear transfer technique is used. Several hypotheses for explaining such a low yield of reprogrammed cells have been put forth:

1) iPSC formation requires specifically narrow ranges of the expression levels of transcription factors. Upon simultaneous transfection of several genes within a lentiviral construct, the distribution of gene expression over cells obeys the probability law (because of different viral copy counts per cell and random integration into the genome). This is presumably the reason why only a small fraction of the transfected cells acquire the “proper” set of expression levels of the reprogramming factors. The pluripotent status of ESCs is known to be quite sensitive to the expression levels of pluripotency genes; e.g., a 50% variation in the Oct4 expression level results in ESC differentiation [44].

2) The populations of reprogrammed somatic cells are heterogeneous and contain a certain amount of cells that are more susceptible to reprogramming than the other cells. For example, some cells (most likely, the dividing ones) during the transfection contain chromatin in a relatively decondensed state facilitating the reprogramming.

3) An unusually high expression level of the exogenous reprogramming factors activates the stress-associated genes that suppress proliferation. Thus, the expression of the *Cdkn1a* and *Cdkn2a* genes, the inhibitors of cyclin-dependent kinases that are involved in various differentiation pathways, increases in the transfected fibroblasts [45]. This fact is explained by the addition of transcription factors, since it is already known that the *Cdkn1a * expression is induced by the Klf4 factors, whereas *Cdkn2a * is activated due to the aberrant expression of *c-Myc * [46]. Thus, the internal self-preservation mechanisms are activated in the transfected cells; these mechanisms suppress uncontrollable proliferation, finally resulting in a low percentage content of cells with a chance of overcoming the barrier of proliferation suppression and achieving the pluripotent state.

4) Insufficient number of reprogramming factors. When using the techniques of reprogramming via cell fusion and nuclear transfer, a somatic cell or its nucleus is subjected to the action of all the components of the pluripotency transcriptional network. These components act at all levels, whereas only a limited number of factors act on the cells upon reprogramming via lentiviral transfection. These factors can activate the transcription cascades only at the very beginning, which makes the reprogramming process more vulnerable to and dependent on random variations.

Based on these hypotheses, one can conclude that the reprogramming induced by the transfection of transcription factors is characterized by a low efficiency and multistageness, as well as being strongly dependent upon stochastic processes. The significance of random variations upon iPSC formation is supported by data showing that the resulting reprogrammed cells are appreciably heterogeneous in terms of the general profile of pluripotency gene expression, epigenetic profile, and morphology. It was demonstrated that the iPSCs originating from the same parental cells reactivate the expression of endogenous *Oct4 * at different time points during the entire reprogramming process, attesting to the multistageness of epigenetic rearrangements and the reprogramming in general [27]. However, when comparing the reprogramming method under consideration with the previously proposed techniques of nuclear transfer and cell fusion, one must allow for the fact that the method proposed by Takahashi and Yamanaka has a number of undeniable advantages, such as a relatively low cost and the simplicity of the reprogramming technique. The universality of the approach should also be noted, since it enables reprogramming of human cells, which had previously been impossible.

## NEW methods for reprogramming somatic cells to a pluripotent state

The original technique for the transfection of reprogramming transcription factors using lentiviral vectors has a number of substantial drawbacks that impede its application in clinical practice. Virus integration into the host genome (up to 20 insertions per reprogramming procedure) increases the risk of tumor formation, since virus incorporation into the target cell genome can accidentally activate or inactivate the host genes, thus increasing the mutagenesis risk. Continuous transgene overexpression is also problematic because of possible incomplete transgene suppression during the reprogramming and upon subsequent differentiation. Even the presence of several pluripotent stem cells in the transplanted tissue may result in tumor development [47].

One of the strategies to solve these problems is based on the reduction of the number of viral vectors introduced, which is achieved via the construction of polycistronic viral vectors carrying several target genes. Constructs encoding the four major reprogramming genes (KMOS) were designed in [47, 48]. The number of viral integrations into the genome was successfully reduced to 3–5 insertions per cell and the homogeneous expression of all four genes in one cell was provided via the use of a single lentiviral construction containing KMOS. Mouse fibroblasts were successfully reprogrammed via the integration of the polycistronic lentiviral vector encoding three genes, *Oct4, Sox2, Klf4* (KOS), followed by the removal of the vector from the genome. This approach enhances the attractiveness of the method due to the fact that the viral material is completely eliminated from the reprogrammed cells [49]. A similar procedure was applied in [50]: a specific piggiBag transposon containing KMOS was used; subsequently, it was eliminated to obtain iPSCs from mouse embryonic fibroblasts, which were free of transgenes and vector sequences. This method has also been used for human cells in some studies [51]. A considerable drawback of the aforementioned approach is that the elimination process of a number of transposons following the reprogramming is difficult to control; moreover, it does not guarantee a 100% result.

The technique for iPSC generation via plasmid transfection of the major pluripotency factors (KMOS or LNOS – *Lin28, Nanog, Oct4, Sox2* ) based on temporary expression of the inserted genes was the next approach in the attempts to resolve the problem of viral integration into the genome. Successful application of this technique for iPSC generation from various cell cultures, including hepatocytes and HEK293 cells, has been reported in a number of studies [52–55]. In the course of improving the technique, the cells were successfully transfected with a single plasmid construct encoding the KMOS canonical gene set. This construct was eliminated from the cells following the reprogramming [54]. It should be noted that one of the drawbacks when using plasmid constructs (compared to the viral vector-based methods) is an extremely low reprogramming efficiency, since this method was mostly used to generate iPSCs from mouse embryonic cells and cell lines known for their lability. However, efficiency in reprogramming human fibroblasts was enhanced by 1% by using riP/EBNA1 episomal plasmid vectors that can encode six genes ( *Oct4, Sox2, Klf4, c-Myc, Lin28* , and *Nanog* ) at once [56].

Another unavoidable drawback of the techniques based on plasmid vectors is the possibility that residual DNA vectors may be present in the target cells after the reprogramming, and therefore the theoretical possibility of insertion mutagenesis [56]. Several methods were designed while searching for approaches that would eliminate the possibility of the incorporation of foreign DNA into the host DNA. Human iPSCs were generated using the Sendai transgenic virus with a reproductive cycle based only on RNAs, which contains neither the stage of DNA reverse transcription (as is the case in lentiviral vectors) nor the stage of integration into the host genome [57]. The advantage of this method is the relatively high gene introduction into various cells and tissues; the drawbacks include the complicated handling of the Sendai virus and the compulsory purification of the reprogrammed cell to remove the replicating virus [57].

Another reprogramming approach without the use of DNA vectors is based on the delivery of reprogramming factor proteins directly into the cells. A specific complex of recombinant proteins consisting of the polyarginine protein-transducing subunit bound to all four major reprogramming factors (KMOS) was designed to create proteins capable of penetrating through the plasma membrane of somatic cells [58]. This approach is relatively simple; the risk of changes in the target cells caused by exogenous genetic sequences decreases when using this technique [58]. However, in a later study in which human cells were used as an object [59], low efficiency was reported for the method. The efficiency of reprogramming using KMOS proteins conjugated to the cell-penetrating peptide (CPP), which contains a large percentage of basic amino acids and is capable of penetrating through the cell membrane, was equal to 0.001%, which is lower than that in the methods based on viral integration by two orders of magnitude.

Transfection of the *in vitro* synthesized mRNA of transcription factors is another promising method for reprogramming somatic cells without the use of DNA vectors. The researchers used mRNA of the LNOS genes [60] to successfully reprogram human neonatal fibroblasts to a pluripotent state. However, despite the fact that the result was achieved, a low reprogramming efficiency (0.0005%) was also observed. The authors attributed this problem to the high cytotoxicity of large mRNA doses [60]. However, the difficulties were overcome via the use of synthetic mRNA of the KMOS and *Lin28* genes, which was comprised of modified ribonucleotides [61]. Combined with the use of the interferon inhibitor B18R and cell culturing under low oxygen content, this technique enabled the attainment of low cytotoxicity and transfection. Thanks to these modifications, the reprogramming efficiency increased by two orders of magnitude and reached 4.4% compared with the 0.04% that was obtained using viral transfection. A large-scale research project focused on the reprogramming of a wide range of somatic cells (including human cells) and analysis of the resulting iPSCs was subsequently carried out [61].

## THE USE OF SMALL MOLECULES FOR REPROGRAMMING

The use of low-molecular-weight compounds, the so-called small reprogramming molecules, is one of the approaches to the reprogramming of human somatic cells. Combined with the earlier designed methods, these molecules are capable of either functional substitution of particular reprogramming factors or facilitating the increase in efficiency of the process. Thus, BIX-01294 (BIX), an inhibitor for the G9a histone methyltransferase, was used. The application of this agent in addition to the transfection using the *Klf4* , *c-Myc* , and *Sox2* , as well as the *Klf4* and *Oсt4* sets within the lentiviral vectors, considerably enhanced (by a factor of 6–10) the yield of the reprogrammed cells [45]. This is attributed to the specific activity of BIX, which facilitates chromatin de-condensing and therefore can functionally substitute the c-Myc transcription factor [45]. 2-propylvaleric acid (valproic acid, VPA) is another compound capable of considerably increasing the reprogramming efficiency [62]. It can specifically inhibit DNA methyltransferases and histone deacetylase. According to [38], the use of this small molecule, in addition to the standard KMOS set, enhances the reprogramming efficiency by 1–2 orders of magnitude and allows one to dispense with the *c-Myc* oncogene. The positive effect of 5-azacytidine (5-azaC) on the yield of reprogrammed cells was demonstrated using the same strategy for DNA methyltransferase inhibition [29]. Reprogramming efficiency can also be increased via the introduction of a small interfering RNA (siRNA), which inhibits the transcripts of the commitment-associated genes [29]. The positive effect of CHIR99021, a specific inhibitor of glycogen synthase kinase 3 (GSK-3), on efficiency in the reprogramming of mouse embryonic fibroblasts has been described. The yield of iPSC colonies was considerably increased by using CHIR99021. The number of reprogramming factors was reduced to two, *Klf4* and *Oct4,* thanks to the use of this reagent in a number of experiments [63].

Small molecules, such as arginine methyltransferase inhibitor AMI-5 and transforming growth factor β inhibitor A-83-01, facilitate the reprogramming process [64]. The induction of mouse fibroblasts by *Oct4* only and the addition of the two aforementioned small compounds resulted in the generation of iPSCs that expressed the typical pluripotency markers and could be differentiated into cells of three germ layers and produce viable chimeric mice. AMI-5 activity is comparable to the joint effect of three components (CHIR99021, Parnate and VPA). AMI-5 inhibits the activity of PRMT 1/3/4/6 and belongs to the family of proteins that catalyze mono- or dimethylated arginine residues. However, it remains to be determined how AMI enhances Oct-4-induced cell programming.

Interesting results were obtained when studying the effect of vitamin C on iPSC generation [65]. It turned out that the treatment of cells undergoing reprogramming with vitamin C in combination with the activation of the *Klf4, c-Myc* , and *Oct4* genes resulted in a considerable decrease in the p53 and p21 levels, as well as in the concentration of reactive oxygen species (ROS). It has been assumed that this factor enhances the reprogramming efficiency, since an increase in the ROS level is usually observed upon transfection with viral vectors. Sodium butyrate has a positive effect on the generation of iPSCs from adult and embryonic human fibroblasts [51]. It is suggested that sodium dutytate promotes the expression of DNA demethylase and H3 acetylation, which ultimately facilitates the expression of endogenous pluripotency factors, including *Oct4* and *Dppa2* (developmental pluripotency associated 2). The screening of various low-molecular-weight compounds was performed in one of the recent studies [66] focused on the role of small molecules in the processes of reprogramming and maintenance of the pluripotent status. A “cocktail” consisting of three molecules, PD98059 (mitogen-activated protein kinase inhibitor), CHIR99021 (glycogen synthase kinase inhibitor), and Y27632 (Rho kinase inhibitor), was selected based on the results of the study. The cocktail demonstrated a considerable effect on the ability of human ESCs to maintain their undifferentiated state under various culturing conditions.

## direct reprogramming of somatic cells

The so-called direct reprogramming can be attributed to areas that require special attention. This strategy presupposes the use of various methods for the transdifferentiation of a specialized cell type into another one, bypassing the stage of formation of pluripotent stem cells. If the method for direct reprogramming is designed, it would be possible to use cell technologies in clinical practice.

Study [67] can be mentioned among such research; in the study, mature exocrine cells from mouse pancreas were *in vivo* reprogrammed into β-similar cells using adenoviral transcription of the genes of three transcription factors, *Ngn3 (Neurog3), Pdx1, * and *Mafa* . The morphology, ultrastructure, expression of the major markers, and key functions (insulin synthesis) of the induced β-cells were identical to those of the intact cells [67]. The data on the influence of the Oct4 transcription factor on the plasticity of mouse keratinocytes were published. Plasmid transfection of the * Oct4* gene was used to obtain a modified cell culture capable of differentiating into neural lineages under certain culturing conditions [68]. This field of research entered its next cycle of development in 2010, when it was demonstrated [69] that a short-term expression of the *Oct4* gene is sufficient in order to change the direction of differentiation of human keratinocytes, including the neural and mesenchymal lineage commitment.

The study where direct reprogramming of embryonic and neonatal mouse fibroblasts was induced *in vitro * [70] is also worthy of mention. A combination of 19 genes specific for the neural tissue and neurogenesis were used to successfully select three genes that perform cell transdifferentiation in the neuronal direction. Retroviruses carrying the *Ascl1, Brn2* , and *Myt1l* genes were used to infect fibroblast cultures and to observe the formation of functional neurons with a complex morphology. It also turned out that the formation of such characteristics of neural cells as the expression of certain neuron-specific voltage-dependent channel proteins, which are required to generate the action potential, can be carried out using a single *Ascl1 * gene. However, joint expression of additional factors is required to make neuronal cell conversion easier and to provide for their complete maturation [70]. A similar result was reportedly obtained using human fibroblasts [71]: the cell phenotype was changed towards dopaminergic neurons after the *Lmx1a* and *FoxA2 * genes were additionally inserted into the cells. It was proposed that astrocytes be used as an alternative source for the generation of cells with the characteristics of dopaminergic neurons [72].

Despite the apparent complexity related to the transdifferentiation of cells derived from one germ layer into cells derived from another germ layer, a number of studies have looked into the problem of cell plasticity. In these studies, evidence attesting to such a possibility was obtained both *in vitro* and *in vivo* [73–75].

Soda *et al* . successfully transdifferentiated glioblastoma cells into endothelial cells [73]. It was demonstrated that glioblastoma cells can be transdifferentiated into vascular endothelium and produce functional blood vessels that are insensitive to the inhibition of the VEGF receptor. The results of this study attest to the existence of a different mechanism of resistance of glioblastoma cells to anti-VEGF-therapy. The reprogramming of terminally differentiated hepatocytes to neuron-like cells has been reported [74].

Results of a successful direct reprogramming of mouse and human fibroblasts towards the neuronal differentiation direction have been published [75]. The reprogrammed cells, in which the expression of the *Ascl1* ( *Mash1), Nurr1,* and *Lmx1a* genes was induced, were very similar to brain dopaminergic neurons in terms of the specific protein production, dopamine release, and pace-making activity. Researchers place their hopes on direct reprogramming of one type of cells to dopaminergic neurons that may be usefull in investigating and treating some neurodegenerative diseases, such as Parkinsons’s disease. 

## THE differences between iPSCs and ESCs. EPIGENETIC “MEMORY” OF iPSCs

Despite the fact that many characteristics of iPSCs are rather similar to those of ESCs, there are also significant differences between these cell types. Among others, there are differences in the levels of control of pluripotency gene expression and in the formation of viable organisms after these cells are transplanted into a developing blastocyst to generate chimeric mice. Evidence has been obtained in support of the fact that the methylation levels of CpG islands in ESCs and iPSCs are similar [76]. A full genome analysis of the CpG islands localized in the functional regions comprising more than 14,000 genes revealed the difference in the methylation levels of 46 genes. The total CpG methylation of the promoter regions in pluripotent cells is higher in comparison to that found in somatic cells. Two ESC and iPSC lines derived from material that was genetically identical to ESCs were compared [77]. In animal chimera experiments, viable mice were successfully obtained from two ESC lines, whereas no animals were obtained from iPSCs. After thorough comparison of the RNA transcript profiles, it was ascertained that the transcription of the imprinted gene cluster *Dlk1-Dio3* in iPSCs is considerably lower than that in the ESC lines. It was detected that the region on chromosome 12 containing the key genes for fetal development was silenced in the iPSC line. Over 60 iPSC-like cell lines were also tested; a similar result was observed in most cases. It should be noted that this gene cluster was activated in a number of iPSC lines. Chimeric living mice were subsequently obtained from these cell lines. Thus, the state of this imprinted cluster allows one to introduce another characteristic for the adequacy of iPSC reprogramming [77]. iPSCs can be differentiated into definitive endoderm precursor cells to design approaches to the cell therapy of damaged tissues of endodermal origin despite the fact that there are some differences between them at the molecular level [78].

It has been assumed that in addition to their potential application for the purpose of regenerative medicine, human ESCs and iPSCs can be used to simulate human inherited disorders. Meanwhile, before using these cells as a model for a particular disease, one needs to assess whether they contain any chromosomal rearrangements, which have put limits on the application of reprogrammed cells. Significant differences between the chromosomal characteristics of iPSCs and ESCs have been revealed [79]. iPSCs were obtained from the skin cells of three patients with a fragile X syndrome (FX) that is responsible for delayed mental development. Unlike ESCs obtained from patients with the FX syndrome, *FMR1* gene expression in certain types of differentiated cells from the same patients was reduced due to anomalous duplications of triplet repeats. It was demonstrated that iPSCs contain a mutated * FMR1* gene, which was not changed in the course of reprogramming, despite the pluripotent status [80]. The study made it apparent that iPSCs are not always suitable candidates for the simulation of diseases associated with epigenetic changes, including imprinting. In a similar study [81], DNA methylation patterns were analyzed in the genomes of 15 cell lines (four ESC lines, five human iPSC lines and the tissues from which these iPSCs were derived, and the differentiated cells obtained from the aforementioned two stem cell lines). Significant differences between iPSCs and ESCs were revealed; the methylation patterns near the chromosome ends and centres of iPSCs remained identical to those in the differentiated cells from which they were obtained. It is clear that reprogramming is a means for acquiring a pluripotent status other than obtaining cells from the embryos. Relying on these data, one can conclude that formation of certain cell types from reprogrammed cells may be restricted. The fact that reprogrammed stem cells have an epigenetic “memory” agrees with the recently published results of a comparison of iPSCs, ESCs, and pluripotent mouse cells obtained using the nuclear transfer procedure [82]. It was demonstrated that iPSCs contain residual epigenetic marks; however, these marks can be eliminated upon continuous culturing or by using specific agents that rearrange the chromatin structure. It was also ascertained that pluripotent stem cells obtained by nuclear transfer reprogram the epigenetic profile more efficiently in comparison with iPSCs.

In addition to the epigenetic “memory,” the existence of gene duplications or deletions associated with genomic instability is the significant comparative characteristic of iPSCs. Genotyping of single nucleotide substitutions was used to compare 69 ESC lines and 37 iPSC lines between, as well as with linear and primary human cell cultures [83]. The results of this thorough study attest to the fact that pluripotent cells in general (and iPSCs to a larger extent) tend to accumulate duplications in the genomic regions containing the pluripotency genes and oncogenes, as well as to accumulate deletions in the region containing tumor growth suppressing genes.

Many researchers attribute the differences between iPSCs and ESCs to the reprogramming procedure and the existence of viral insertions into the genome. The transcription profiles of human ESCs and iPSCs were compared using methods without the use of viral constructs [84]. The transcription profiles of ESCs and iPSCs were shown to be largely similar; however, some differences were detected, which cannot be attributed to viral integration into the genome.

## THE POTENTIAL OF USING iPSCs in CLINICAL PRACTICE

Allogenic organ transplantation is associated with a number of problems, such as limited tissue engraftment and the necessity to use immunosuppressors. It is believed that these problems can be overcome by reprogramming the patient’s own cells, because the cells grafted to the recipient will be genetically identical to his own cells. The method proposed is undoubtedly superior to the existing transplantation techniques because of the possibility of *in vitro* study and repair of the pathological mutations in the cells. For example, sicklemia has been successfully repaired using iPSCs on a mouse model [85]. The formation of normal erythrocytes from hematopoietic precursor cells obtained from completely reprogrammed skin cells was observed in [85].

Many diseases, such as diabetes mellitus type 1, the Parkinson’s and Alzheimer’s diseases, etc. are very difficult to study and cure both because the damaged organ is difficult to reach (hence the difficulties associated with the search for donor tissue) and because no methods have been designed for the continuous cultivation of the proper cell lines. When simulating these disorders, autological iPSCs can be obtained, followed by their differentiation in a culture into the required cell line to produce adequate test systems for the screening of pharmaceutical agents. These test systems can also be used to investigate the diseases accompanied by pathological motoneuronal death (e.g., in patients with amyotrophic lateral sclerosis or spinal muscular amyotrophy). The lack of cell materials originating from patients at late stages of the development of a disease is one of the main problems associated with the study of degenerative pathologies. Since iPSCs presumably have to undergo all the differentiation stages *in vitro,* same as the recipient’s cells before the disease develops *in vivo* , this technology can make it possible to study the early stages of a particular disease. Active research has been carried out; iPSCs have been already obtained in some laboratories from patients with Hungtington’s disease, sicklemia, myodystrophy, the Down’s syndrome, etc. [13, 14, 86, 87].

Considerable differences between the same cell types differentiated from ESCs and iPSCs have been revealed [88]. Study of teratoma formation in C57BL/6 and 129/SvJ mice has demonstrated that the disruption of gene expression in some cells differentiated from iPSCs may result in a T cell-dependent immune response in an isogenic recipient. Thus, the currently available reprogramming technologies are still a long way from clinical application. One of the primary tasks consists in the design of methods that would enable the epigenetic differences between iPSCs and ESCs to be minimized.

## APPROACHES TO THE CLINICAL USE OF iPSCs

The use of oncogenes to obtain iPSCs is one of the major problems impeding the therapeutical use of these cells. The *с-Myc* oncogene is hyperexpressed in approximately 70% of human tumors; therefore, the hyperexpression of an inserted transgene makes the use of iPSCs dangerous [89]. In order to solve this problem, iPSCs obtained from humans and mice were subjected to study. No postnatal tumor development was observed in chimeric mice obtained from iPSCs without introduction of *c-Myc* , whereas oncological diseases developed in ~15% of the animals obtained from iPSCs with exogenous *c-Myc * [90]. *Oct4, Sox2* , and * Klf4 * can also be associated with the emergence of different types of tumors; therefore, researchers increasingly try to avoid the transduction of these oncogenes [54, 56, 61, 91]. In order to achieve the necessary results, target cells are selected that would endogenously express the required factor at an adequate level, hence its introduction would be rendered unnecessary. Thus, the endogenous *Sox2 * gene is strongly expressed in neutral stem cells; these cells were successfully reprogrammed in a number of experiments by inserting *Oct4 * and *Klf4* only [45, 92] or even *Oct4 * alone [92, 93]. Meningiocytes and keratinocytes can be regarded as promising cells for reprogramming because of their relatively high levels of *Sox2* [94], *c-Myc, * and * Klf4* [95, 96] expression. It has also been discovered that it is easier to derive iPSCs from amniotic fluid cells because of the fact that they are relatively weakly differentiated [97, 98]. The rate of iPSC formation from amniotic fluid cells is at least twice faster than that of iPSC formation from fibroblasts, whereas the reprogramming efficiency in the former case is higher by an order of magnitude. One of the approaches to reprogramming consists in the replacement of oncogenes for small molecules [38, 45]. Teratogenicity of iPSCs is a significant issue, since there may remain a certain amount of undifferentiated iPSCs that are dangerous for the recipient after these cells are differentiated into the specialized cells intended for transplantation [99]. The search for selection methods that would ensure the isolation of iPSCs from the differentiated cells continues. The karyotypic instability of pluripotent cell lines has been revealed via the study of the chromosome composition of ESCs and iPSCs [100], attesting to the necessity for a thorough cytogenetic analysis of iPSCs and initial cell lines.

The similarities and differences between ESCs and iPSCs are being actively investigated at the molecular and functional levels. The results of these studies may influence the therapeutic applicability of iPSCs. This field of research (as well as the development and optimization of differentiation protocols and the establishment of reliable criteria for the application of specialized cells generated from iPSCs) requires an analysis of the genomic and epigenomic statuses of human iPSCs. 

## Abbreviations

ESCs: embryonic stem cells
iPSCs: induced pluripotent stem cells
Chd1: chromodomain helicase DNA-binding protein
BAF: rg/Brahma-associated factors
miRNA, miR: microRNA
TERRA: telomeric-repeat-containing RNA
Cdkn: cyclin-dependent kinase inhibitor
VPA: valproic acid
siRNA: small interfering RNA
KMOS: Klf4, c-Myc, Oct4, Sox2
KOS: Klf4, Oct4, Sox2
LNOS: Lin28, Nanog, Oct4, Sox2
GSK-3: glycogen synthase kinase 3
ROS: reactive oxygen species
HIF1: hypoxia-inducible factor 1
VEGF: vascular endothelial growth factor
